# Learning from errors? The impact of erroneous example elaboration on learning outcomes of medical statistics in Chinese medical students

**DOI:** 10.1186/s12909-022-03460-1

**Published:** 2022-06-17

**Authors:** Chengwei Wang, Junyi Li, Haiyan Li, Yijing Xia, Xiaoyu Wang, Yufei Xie, Jinyang Wu

**Affiliations:** 1 Department of Integrated Traditional and Western Medicine, West China Hospital, Sichuan University, Chengdu, China; 2 College of Psychology, Sichuan Normal University, Chengdu, China; 3 Department of Neurosurgery, West China Hospital, Sichuan University, Chengdu, China; 4 Department of Orthodontics, Shanghai Xuhui District Dental Disease Prevention and Control Institute, Shanghai, China; 5 Department of Oral and Cranio-maxillofacial Surgery, Ninth People’s Hospital, Shanghai JiaoTong University School of Medicine, Shanghai, China; 6 College of Stomatology, Shanghai Jiao Tong University, Shanghai, China; 7 Shanghai Key Laboratory of Stomatology & National Center for Stomatology & National Clinical Research Center for Oral Diseases, Shanghai, China

**Keywords:** Erroneous example, Elaboration training, Metacognitive load, Medical statistics, Academic performance

## Abstract

**Background:**

Constructivism theory has suggested that constructing students’ own meaning is essential to successful learning. The erroneous example can easily trigger learners’ confusion and metacognition, which may “force” students to process the learning material and construct meaning deeply. However, some learners exhibit a low level of elaboration activity and spend little time on each example. Providing instructional scaffolding and elaboration training may be an efficient method for addressing this issue. The current study conducted a randomized controlled trial to examine the effectiveness of erroneous example elaboration training on learning outcomes and the mediating effects of metacognitive load for Chinese students in medical statistics during the COVID-19 pandemic.

**Methods:**

Ninety-one third-year undergraduate medical students were randomly assigned to the training group (*n* = 47) and the control group (*n* = 44). Prerequisite course performance and learning motivation were collected as covariates. The mid-term exam and final exam were viewed as posttest and delayed-test to make sure the robustness of the training effect. The metacognitive load was measured as a mediating variable to explain the relationship between the training and academic performance.

**Results:**

The training significantly improved both posttest and delayed-test performance compared with no training (*F*_*posttest*_ = 26.65, *p* < 0.001, Partial *η*^*2*^ = 0.23; *F*_*delayed test*_ = 38.03, *p* < 0.001, Partial *η*^*2*^ = 0.30). The variation trend in metacognitive load in the two groups was significantly different (*F* = 2.24, *p* < 0.05, partial *η*^*2*^ = 0.20), but metacognitive load could not explain the positive association between the treatment and academic performance (*β* = − 0.06, *se* = 0.24, *95% CI* − 0.57 to 0.43).

**Conclusions:**

Erroneous example learning and metacognitive demonstrations are effective for academic performance in the domain of medical statistics, but their underlying mechanism merits further study.

## Introduction

Medical statistics is a compulsory course for medical students at all grade levels in China. It mainly focuses on summarizing, collecting, presenting, and interpreting medical practice data and using them to estimate the magnitude of associations and to test hypotheses. To learn medical statistics well, students should recall the content of textbooks and fully understand the principles of statistics, construct their knowledge framework, and internalize what they have learned based on their own experience and insights [1]. Constructivism believes that learning is not a process of passive absorption, repeated practice and memory strengthening but rather a process of actively constructing meaning through an individual-environmental interaction (i.e., assimilation and accommodation) based on the existing knowledge and experience of the students [2]. Understanding learning material and constructing students’ own meaning are vital to statistical learning or the learning of any other discipline. Elaboration processes are essential for meaningful learning since they allow learners to organize knowledge into a coherent structure and integrate new information with existing knowledge structures [3]. Students do not come to class as “empty vessels” waiting to be filled but instead approach learning material with significant prior knowledge. They need to interpret the new material in terms of their knowledge [1]. Thus, the instructional design of medical statistics should be improved by helping students construct their own meaning for what they are learning.

### Worked-out example learning

Existing studies have suggested that the worked-out example is an efficient and effective instructional tool [4]. The worked-out example (i.e., consisting of a problem formulation, solution steps, and the final solution) has proven successful in various domains. Cognitive science suggests that seeing worked-out problems first makes the task easier and leads to greater understanding in less time [5]. Worked-out examples are problems that are completely worked out, showing all the steps of a solution. Using self-explanation, students decide why those steps are correct. Furthermore, when students can explain to themselves about why and how the correct answer is obtained, they gain a greater mental image of the process in different problem situations [6]. Cognitive psychology provides evidence that using worked-out examples and delaying actual problem-solving practice benefits novice learners [7]. For instance, in an introductory statistics class, students who studied worked-out problems demonstrated better academic performance on different statistical concepts [8]. Additionally, learners’ active self-explanations of worked-out examples lead not only to enhanced near transfer but also to better far transfer [9]. This shows that the worked-out example plays a vital role in learning transfer. Without worked-out examples, students do not understand formulas properly and thus cannot apply them.

However, the adoption of a worked-out example is no panacea. The benefits that learners can obtain from it are not as significant as we thought. There is evidence that worked-out examples may be more suitable for novice learners [10]. The theoretical basis behind this is that in worked-out examples, learners will be more inclined to perform shallow processing, making it challenging to conduct deep learning and elaborate [11]. The extent to which learners benefit from the worked-out example depends heavily on how well they explain the solutions of the example to themselves [12]. Worked-out examples also vary in effectiveness depending on learner characteristics (especially prior knowledge) and on the learning outcomes considered [13]. Researchers have found that many learners are passive or superficial explainers; they exhibit a low level of elaboration activity and spend little time on each worked-out example. Passive and superficial example elaboration spontaneously occurred more frequently than deep elaboration [14]. This is probably because the solution steps and the final solution have already been provided to the students, which may easily give them the illusion of understanding. Namely, the given steps and correct answers may be detrimental to students’ metacognitive monitoring and further affect learning outcomes.

### Erroneous example learning

Why do we not provide students with an erroneous example to learn from instead of a correct example? An erroneous example is a worked example that incorporates at least one incorrect solution step [15]. It contains a common, well-documented misconception in a particular domain, a few self-explanation prompts/hints, and the correct solution [16]. These components form a scaffolding so that learners can identify errors, correct the erroneous problem-solving steps, and better use their abilities to generate solutions and solve problems correctly [17]. Experienced teachers can determine which parts of the learning material are prone to mistakes and can then compile erroneous examples, allowing students to see these examples, find the mistakes, and explain and correct them [18]. In this way, erroneous examples push students to further deepen their understanding of the content, help learners consolidate the concepts, methods, and skills they have learned, and improve learners’ problem-solving and application abilities [19]. In addition, these examples can easily trigger learners’ confusion, which has been proven to precede successful learning [20] because states of uncertainty and confusion may “force” students to deeply process the learning material. Learners are consistently assimilating new information into their prior knowledge during the complex learning task. Deep learning occurs when there is a discrepancy in the information stream, and the discrepancy is identified and corrected [21]. As the discrepancy-reduction model suggests, learners will allocate more study time to difficult learning items (i.e., larger discrepancy between the current perceived state and the learning goal) than easier ones [22]. However, confusion must be effectively resolved by the learner, as unresolved confusion may have adverse consequences for learning [21].

Although some researchers worry that studying erroneous examples might appear to risk reinforcing students’ misconceptions or introducing an inaccurate understanding, exploring students’ errors can play a critical pedagogical role in teaching discussions [23]. There is evidence that the hypothetical errors of others can foster reflection, helping students recognize and correct errors in their own work [20]. This result seems to contradict the belief that showing students incorrect examples may reinforce existing misconceptions or introduce new errors, especially when the teaching materials identify the errors in the examples.

### Metacognitive load and elaboration

Similar to learning worked-out examples, students need to be prompted to identify, explain, and correct the errors during erroneous example learning, which requires a high level of metacognitive monitoring and regulation. According to cognitive load theory (CLT), when learners invest effort in the construction and storage of schemata (e.g., in the process of erroneous example elaboration), they undertake a high level of germane cognitive load (i.e., the load imposed by cognitive processes directly relevant for learning) [24]. However, they also need to invest effort in monitoring this learning activity, which was called “metacognitive load” by Valcke [25]. Schwonke [26] believes that metacognitive load may be directly related to learning activities and learners’ interaction. In the process of learning erroneous examples, students have to consistently monitor the discrepancy between the learning materials (i.e., erroneous examples) and their prior knowledge, and then they regulate their understanding/knowledge structure. Therefore, in the present study, students’ metacognitive load can be used as a potential mediating variable to explain the impact of erroneous examples on learning outcomes. Of course, erroneous example learning may be subject to passive or superficial explanations as well, and many students benefit less from this learning method [12]. According to instructional scaffolding theory and constructivist theory, when students cannot use certain knowledge and skills on their own, they can acquire new knowledge and skills through interaction with teachers [27]. Thus, a training procedure that aims to improve elaboration quality (especially metacognitive load) could be implemented. The instructors would act as a model and demonstrate metacognitive elaboration (e.g., self-explanation prompts, think aloud) utilizing a simple erroneous example. Then, the students would apply the elaboration behaviors demonstrated by the model. In this activity, teachers guide the teaching and enable students to master, construct, and internalize the behavior refined by the model to perform higher-level metacognitive activities and to increase the metacognitive load of students. The ultimate goal is to transfer the responsibility of a learning item to the student through scaffolding (i.e., elaboration training), while support fades over time [28]. We designed learning materials and elaboration training procedures based on previous research [13] and anticipated that the students who received the training would exhibit a higher metacognitive load and achieve better academic performance.

Based on the literature mentioned above, the present study explored the effectiveness of erroneous example elaboration on Chinese students’ learning in medical statistics and the mediating effect of metacognitive load. The state of metacognitive load was examined across different conditions (i.e., without elaboration training vs. with elaboration training) over time. We proposed the following hypotheses:

1. The posttest performance of the experimental group (i.e., erroneous example learning with elaboration training) would be significantly higher than that of the control group.
2. The academic performance of the control group would decrease significantly from posttest to delayed test after retreating the erroneous example learning, whereas this difference would not be found in the experimental group.
3. The metacognitive load would explain the higher performance of the experimental group. Namely, the mediating effect of metacognitive load on the relationship between erroneous example learning and academic performance would be significant.

## Methods

### Participants and design

Ninety-one third-year undergraduate medical students were enrolled in a medical statistics course. Because of the impact of COVID-19, this course was divided into two parts. All students received medical statistics (Part 1) online from March 2020 to July 2020. After the COVID-19 situation alleviated in China, they returned to school and continued to learn medical statistics (Part 2) from September 2020 to January 2021. Ethics approval was granted by the West China hospital Sichuan University Institutional Human Research Ethics Committees (Verified in 2019 - No. 489). All students gave their informed consent before study inclusion.

Medical statistics is a compulsory course for the participants. Its content includes types of data, descriptive statistics for categorical data and continuous data, probability distributions, parameter estimation, hypothesis testing for categorical data and continuous data (e.g., t-test, ANOVA, ANCOVA, and Mann–Whitney U test), measures of association (e.g., Pearson’s correlation coefficient), clustering analysis, simple linear regression, multiple regression analysis, data visualization, and application of statistical software (SPSS). Medical statistics (Part 2) mainly focuses on the content beyond hypothesis testing for categorical data. The course schedule consisted of 16 classes (once per week), and each class lasted 90 minutes.

Forty-four students were randomly assigned to the control group, in which the students were given a series of erroneous example items to engage in self-reflection (i.e., elaboration without training) in the in-class exercise section. After the students received their erroneous example items, the instructors demonstrated one or two worked-out examples on the blackboard (different from the erroneous example students received) relevant to the knowledge component of a given course. This procedure ensured that the two groups received instructors’ guidance before the students explained the erroneous examples themselves. The intervention group included 47 students who were also asked to study erroneous examples in the coursework section. However, the instructors would act as a model and demonstrate metacognitive elaboration (e.g., self-explanation prompts, think aloud) utilizing a simple erroneous example before the students attempted self-explanations themselves. Then, the students applied the elaboration behaviors demonstrated by the model.

### Erroneous examples

**Fig. 1:**
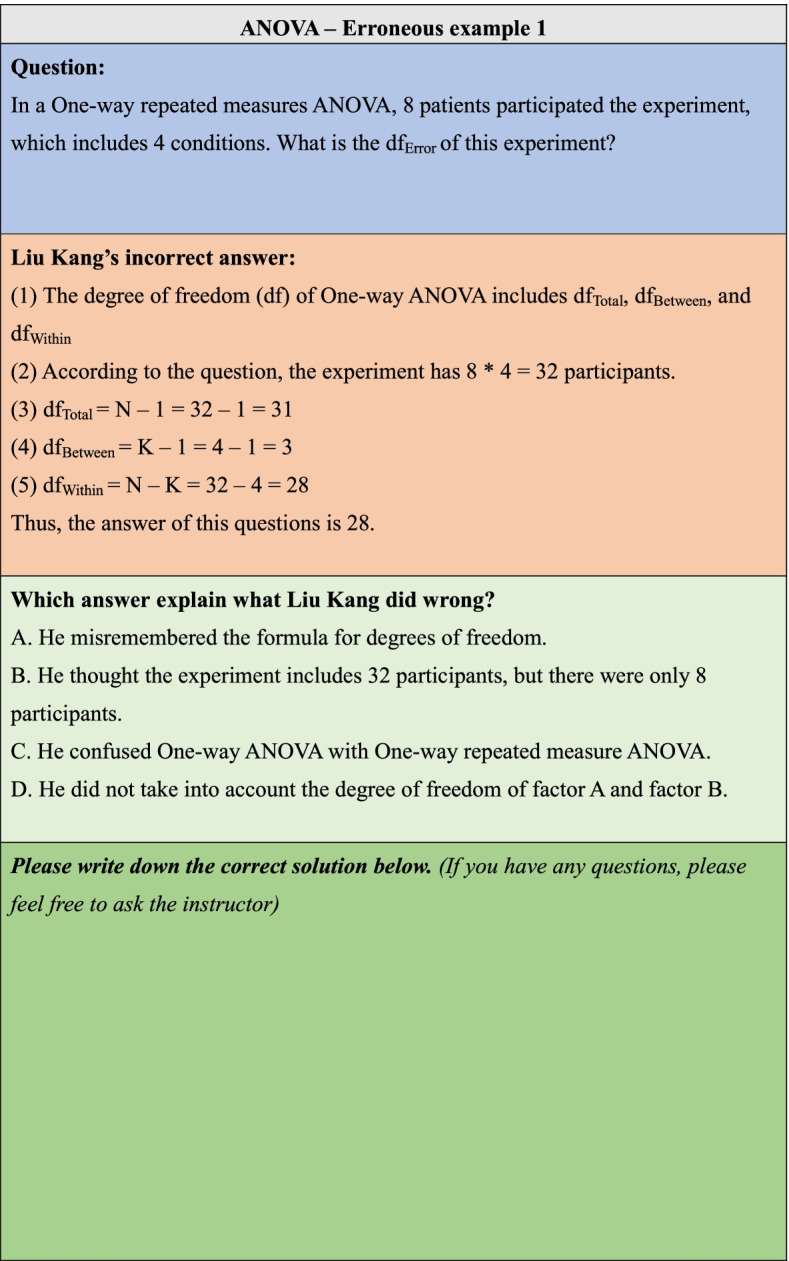
Example of an erroneous example learning item

As shown in Fig. 1, each erroneous example item comprised four parts: question, incorrect answer, explanation of the error, and the student’s answer. Students were informed that the solution was incorrect, and they were asked to study these materials and explain the error. Incorrect solutions often contain one or more common misconceptions relevant to a specific knowledge component. Students needed to respond to a multiple-choice question where they explained the hypothetical student’s error. This kind of question was designed to encourage self-explanation. Instructors provided feedback if the students had any questions.

### Measures

#### Prerequisite course performance

Medical statistics (Part 1) was considered a prerequisite course and included in the model as one of the covariates.

#### Learning motivation

We designed 11 items to assess students’ learning interest in the course as one of the covariates. Some of the learning interest items were adapted from Marsh et al.’s academic interest scale [29]. The items were “I enjoy working on statistical problems”, “Medical statistics is one of the things that is important to me personally”, “I would even give up some of my spare time to learn new topics in medical statistics”, and “When I’m working on medical statistical problems, time sometimes seems to fly by”. The internal consistency of the learning interest scale was 0.73.

#### Metacognitive load

Participants’ subjective mental effort during in-class exercises (i.e., erroneous example learning) was assessed on a 5-point Likert scale. It was recorded 10 times (once per week). After each in-class exercise, the participants scored the amount of mental effort they expended in monitoring/regulating their cognition/explanation/understanding of the erroneous examples. We used this kind of self-rating measurement because it has been demonstrated that people are quite capable of giving a reasonably accurate numerical indication of their perceived mental burden [30]. Studies have also shown that reliable measures can be obtained with a unidimensional scale, are sensitive to relatively small differences in cognitive load and are valid, reliable, and unintrusive [31].

#### Tests

To evaluate the effects of erroneous example elaboration with training, we recorded the participants’ mid-term and final exam scores. All participants received erroneous example learning until the mid-term exam. Thereafter, erroneous example learning was not included in the curriculum. Thus, the prerequisite course performance, mid-term exam, and final exam were considered the pretest, posttest, and delayed test, respectively.

### Procedures

At the beginning of the semester, we required all students to complete the learning interest scale, and their medical statistics (Part 1) performance was recorded as a pretest score. Before the mid-term exam, they learned erroneous examples during in-class exercises (once per week) for 10 weeks. In each week of erroneous example learning, students evaluated their metacognitive load as related to understanding or explaining the examples.

Forty-four students were randomly assigned to the control group (i.e., elaboration without training), and 47 students were randomly assigned to the experimental group (i.e., elaboration with training). The two groups shared the same instructor team, learning materials, classroom, and schedule except for the erroneous example learning method. After learning for 10 weeks, all students participated in the mid-term exam (considered posttest) and continued to learn the remaining chapters (but without erroneous example learning). At the end of the semester, the final exam score was considered the delayed test score.

### Data analysis

First, an unpaired t-test was used to examine the differences in baseline scores (i.e., learning motivation and prerequisite course performance) between the two groups. Second, one-way ANCOVA was conducted to test the significant differences in the outcomes between the two groups using prerequisite course performance and learning motivation as covariates. Third, we conducted a paired-sample t-test to examine the robustness of the effect of elaboration training (i.e., the difference between the posttest and delayed-test for each group). Fourth, repeated-measures ANOVA was chosen to test whether the metacognitive load significantly changed as students worked through erroneous examples. Fifth, the mediating effect of metacognitive load was examined by setting the group (i.e., control vs. experimental group) as the independent variable, posttest performance as the dependent variable, and prerequisite course performance and learning motivation as covariates. All data analysis and data cleansing procedures were conducted by using SPSS 20.0 and the PROCESS macro for SPSS [32].

## Results

As shown in Table 1, the prerequisite course performance and learning motivation were equivalent between the two groups.

**Table 1:** The baseline scores of the learning motivation and prerequisite course performance (Independent sample t-test)

Outcomes	Elaboration without training (*n* = 44)	Elaboration with training (*n* = 47)	*t*	*p*	Cohen’s *d*
Learning motivation	28.70 (4.92)	29.77 (4.08)	1.12	0.27	0.24
Prerequisite course performance	72.89 (5.53)	73.17 (5.80)	0.24	0.81	0.05

Table 2 presents the results of one-way ANCOVA. We considered learning motivation and prerequisite course performance as covariates. The results showed that erroneous example elaboration training significantly enhanced academic performance (i.e., mid-term exam and final exam scores). The participants in the experimental group reported a significantly higher metacognitive load than those in the control group.

**Table 2:** The effects of elaboration training on outcome variables (Learning motivation and prerequisite course performance were controlled as covariates)

Outcomes	Elaboration without training (*n* = 44)	Elaboration with training (*n* = 47)	*F*	*p*	Partial *η*^*2*^
Meta-cognitive load (Mental effort)	27.82 (4.99)	30.34 (4.23)	5.74	0.02^*^	0.06
Mid-term exam score	74.25 (3.72)	78.02 (3.16)	26.65	0.00^***^	0.23
Final exam score (Delayed test)	72.52 (3.53)	77.49 (3.87)	38.03	0.00^***^	0.30

Note: *p < 0.05, ***p < 0.001

We conducted a paired-sample t-test to examine the robustness of the effect of elaboration training (See Table 3). After retreating with erroneous example learning, the academic performance of the control group declined significantly from the mid-term exam to the final exam but with a relatively small effect size (Cohen’s *d* = 0.48). In the experimental group, a significant difference in academic performance between the mid-term exam and the final exam was not found, suggesting that the effect of erroneous example elaboration training was robust.

**Table 3:** The robustness of elaboration training effect from mid-term to the end of term

	Mid-term exam score	Final exam score (i.e., delayed test)	*t*	*p*	Cohen’s *d*
Elaboration without training (*n* = 44)	74.25 (3.72)	72.52 (3.53)	4.48	0.00^***^	0.48
Elaboration with training (*n* = 47)	78.02 (3.16)	77.49 (3.87)	1.12	0.27	0.15

Note: ***p <0.001

**Fig. 2:**
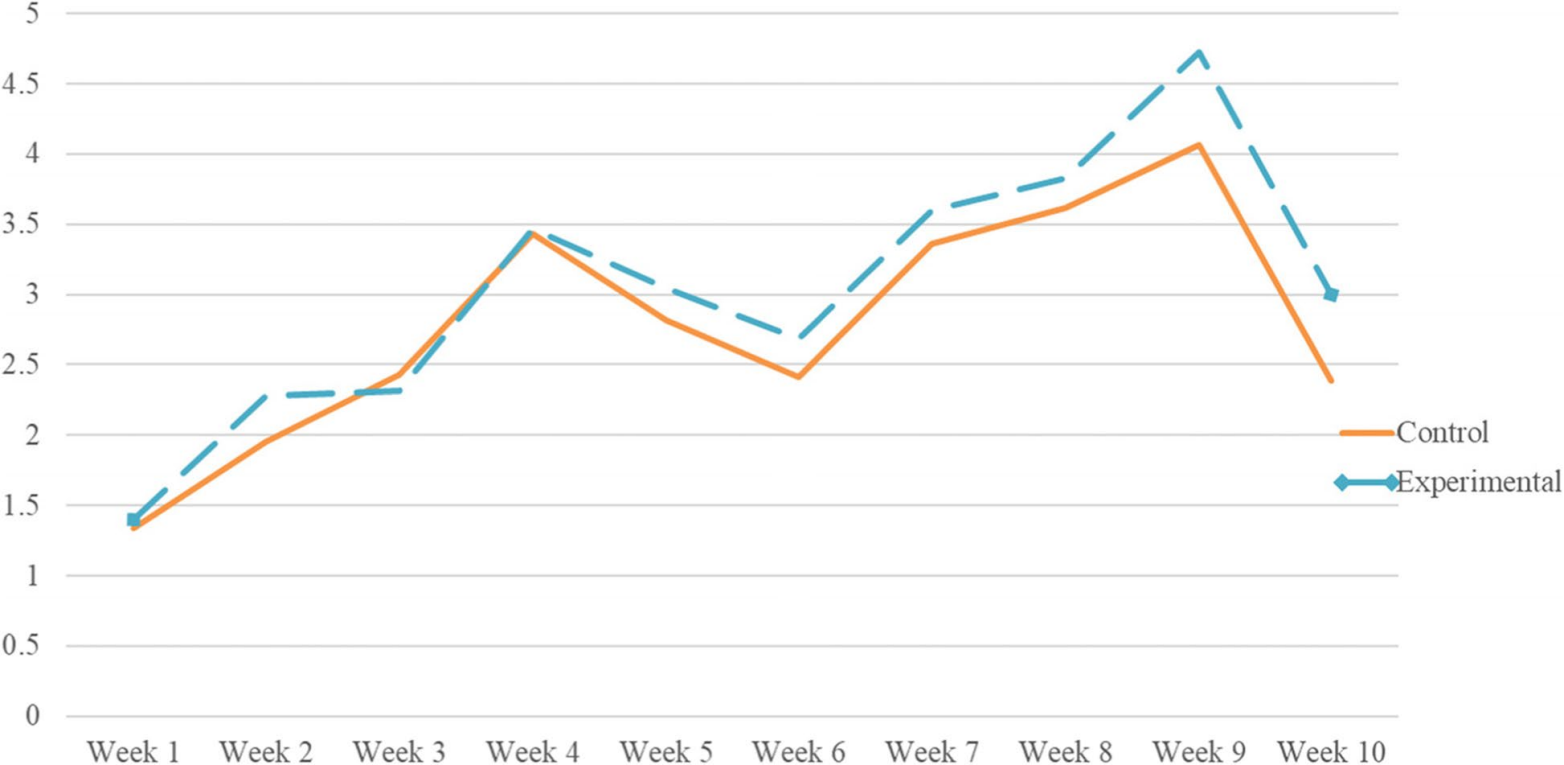
The change of metacognitive load in two groups during the erroneous example learning

Repeated measures ANOVA was performed with metacognitive load as the within-subject factor, group as the between-subject factor, and learning motivation and prerequisite course performance as covariates. We found that both the main effect of the group (*F* = 2.77, *p* < 0.01, partial *η*^*2*^ = 0.24) and the interaction effect between metacognitive load and the group were significant (*F* = 2.24, *p* < 0.05, partial *η*^*2*^ = 0.20), indicating that the variation trend in the two groups was significantly different. This was especially apparent after the fourth round (see Fig. 2), where the metacognitive load of the experimental group was much higher than that of the control group.

The mediation analysis results (Model 4 in the PROCESS macro) showed that the indirect effect of the group on posttest performance was not significant (*β* = − 0.06, *se* = 0.24, *95% CI* − 0.57 to 0.43), indicating that the mediating effect of metacognitive load was not found.

## Discussion

This study examined the effectiveness of erroneous example elaboration training on Chinese students’ learning outcomes in the domain of medical statistics. Metacognitive load was regarded as a possible mechanism to explain the positive effect of erroneous example elaboration training on academic performance. The main findings are as follows: 1). Erroneous example elaboration training significantly improved both posttest and delayed-test performance compared with no training; 2). The effect of treatment was robust; 3). The variation trend in metacognitive load between the two groups was significantly different; the metacognitive load of the experimental group was much higher than that of the control group after the fourth round; and 4). The mediating effect of metacognitive load on the association between group and academic performance was not found.

### Elaboration training and erroneous examples learning

As anticipated, learners in the experimental group exhibited higher posttest and delayed-test performance than those in the control group. Additionally, the academic performance of the control group decreased significantly from the posttest to the delayed test after retreating with erroneous example learning, whereas this significant difference was not found in the experimental group, suggesting the robustness of the treatment effect. Consistent with our results, Stark et al. [33] implemented short elaboration training for apprentices of a bank who studied worked-out examples and found that participants with elaboration training exhibited both deeper elaboration and active metacognitive elaboration. The enhanced elaboration activities further improve learning outcomes.

In the experimental group of the present study, students were provided not only with well-designed erroneous examples but also with instructors’ demonstrations. The instructor modeled how to set subgoals (i.e., planning) and think aloud (i.e., monitoring) and provide self-explanation prompts (i.e., monitoring and evaluation) and self-regulation (i.e., regulation). These metacognitive components were presented to the students but not to controls as a well-structured “package”. Students in the experimental group were more likely to achieve better performance than with learning erroneous examples alone, even with instructors providing feedback when required. After retreating with erroneous example learning (i.e., from week 10 to week 16), students in the two groups received the same instructional procedures, but the academic performance of the control group significantly declined. It is likely that since students without elaboration training or demonstrations had more passive and shallow elaboration activities, they did not form a solid knowledge structure during the first 10 weeks. The performance of the experimental group decreased as well but did not reach significance, possibly because the final exam covered more content than the mid-term exam.

In addition, erroneous example learning seems to improve students’ grades because both groups’ mid-term exam and final exam scores were higher than those of medical statistics (Part 1). This result is consistent with those of previous studies. For example, Zhang found that in medical diagnostic knowledge learning, erroneous examples significantly improved the diagnostic ability of medical students [19]. Researchers have found that learners can deepen their understanding and application of knowledge in the process of interpreting correct and incorrect information [34]. Moreover, feedback can promote student learning [35]. The instructional design of erroneous example learning in both groups of the current study included immediate feedback provided by instructors; most students’ confusion could be resolved in a timely manner rather than remaining stuck in the students’ knowledge structure. Through feedback, learners can know whether they corrected the erroneous example themselves the very first time they interpreted it. It is helpful for students to grasp, understand and consolidate correct knowledge in a timely manner [18]. Metcalfe reviewed behavioral and neurological research and found that mistakes can greatly facilitate new learning [36]. Partially because the state of confusion focuses students’ attention on discrepancies, it signals a need to initiate intensive deliberation and problem-solving processes. It also influences knowledge restructuring when impasse resolution or misconception correction leads to the reorganization of an incomplete or faulty mental model [21]. Richey et al. also suggested that students learn more from erroneous examples than from the problem-solving condition in an intelligent tutoring system [13]. Erroneous examples enhance the memory and generation of correct answers in the future, promote active learning, arouse learners’ attention, and inform learners of error-prone knowledge points [36]. Thus, teachers should be encouraged to be open to mistakes and actively use erroneous example learning in instructional design to facilitate students’ learning.

### Metacognitive load is not the underlying mechanism

We initially expected that the difference in metacognitive load between the two groups may explain the higher academic performance of the experimental group. However, there was no mediating effect of metacognitive load on the association between group and academic performance. We found that the metacognitive load trajectories of the two groups were significantly different. After the fourth round, the metacognitive load of the experimental group was much higher than that of the control group. Namely, students in the experimental group invested more mental effort in monitoring/regulating their cognition of the erroneous examples. Perhaps the demonstration given by the instructors in the experimental group was more likely to elicit students’ metacognitive load than those in the control group. Although metacognition increased, this mental effort does not seem to translate into academic performance. First, this finding might be because the complex learning material (i.e., erroneous examples) and demonstration in the experimental group pose vast cognitive and metacognitive demands on students. All these demands compete for limited mental resources. They may sometimes be beneficial, sometimes neutral, and occasionally detrimental to learning [26]. In the present study, these metacognitive demands on students exhibited neutral learning. Second, perhaps metacognitive knowledge/beliefs and the regulation/control of cognitive actions are more predictive than metacognitive load. Metacognitive load was assessed by a self-report scale, which is highly questionable as a source of data because people have no direct access to their mental processes [37]. In summary, erroneous example learning and metacognitive demonstration are effective for improving academic performance, but the underlying mechanism deserves further study.

### Limitations and future studies

Some limitations of the present study should be noted. First, we did not measure metacognitive knowledge/beliefs and regulation, which may be an important psychological mechanism. Second, use of the self-report scale to measure metacognitive load is questionable. Fine-grained data such as think-aloud data or log files in an intelligent tutoring system are more feasible. Third, the sample size in each group was relatively small; thus, we may not have acquired enough statistical power and may have further influenced the robustness of the current study results. Finally, our study did not design a group that used the worked-out example. The results of the current study may not be able to effectively prove that the effect of erroneous example learning is better than that of worked-out example learning.

Despite these limitations, our research still provides empirical evidence of applying erroneous example learning methods in medical statistics, proving that erroneous example elaboration training is an effective instructional design. Future research can refine the specific training process on this basis to improve training effectiveness and to develop effective long-term strategies, such as presenting both erroneous examples and worked-out examples in the same workbook and conducting a step-by-step problem-solving exercise. Simply exposing students to incorrect examples may not be enough to improve learning, as students may not understand what makes the error wrong [38]. Thus, it may be necessary for students to have sufficient scaffolds when learning from erroneous examples, especially if they do not have prior in-depth knowledge [39, 40]. Second, future research should include a control group that uses worked-out examples and elaboration training to compare the learning effects of the two examples (i.e., worked-out example vs. erroneous example), refute the deficiencies of the erroneous example, and further explore the advantages of the erroneous example in the field of learning. Third, future studies could collect qualitative data such as survey comments and interviews to further examine learners’ metacognitive load. The application of erroneous examples to multiple disciplines and fields to improve the generalizability of its learning effects is also a promising direction. As Metcalfe [36] wrote, an unwarranted reluctance to engage with errors may have held back our education. Encouraging educators and students to be open to mistakes is an important step to facilitate learning.

## Data Availability

The datasets generated and analyzed during the current study are not publicly available due to ethical and privacy considerations but are available from the corresponding author on reasonable request.
